# A case of squamous cell carcinoma occurring on a scar of Buruli ulcer in Bouake, Ivory Coast

**DOI:** 10.11604/pamj.2019.33.246.19341

**Published:** 2019-07-23

**Authors:** Almamy Diabaté, Koffi Kouamé Pacôme Gbandama, Amon Anderson Stephen Kouabenan, Irenée Gué, Bamba Vagamon, Boussou Romain Aka

**Affiliations:** 1 Department of Dermatology, University Hospital of Bouake, Bouake, Cote d'Ivoire; 2 Department of Dermatology, University Hospital of Treichville, Abidjan, Cote d'Ivoire

**Keywords:** Buruli ulcer, scar, squamous cell carcinoma

## Abstract

Buruli ulcer is infectious necrotizing panniculitis due to *Mycobacterium ulcerans*. Buruli ulcer is healed by leaving dystrophic, fibrous and retractile scars. On these scars can occur long-term squamous cell carcinoma. We report the first case of squamous cell carcinoma occurring on healing of Buruli ulcer. A 32-year-old woman with Buruli ulcer who has been cured for about 10 years is seen for ulcero-bulging knee swelling. The examination had revealed a large swelling of about ten centimeters in diameter, ulcero-budding, with an easily bleeding cauliflower appearance. The diagnosis of squamous cell carcinoma being retained without metastasis, resection of the tumor with scarring after one month without chemotherapy. There was no recurrence after six months of decline. The epidemiology of Buruli ulcer, responsible for scarring, explains the young age of our patient and the localization of carcinoma on the limb. The carcinomatous degeneration of scars, especially the scars of old burns, is constantly reported. The characteristics of Buruli ulcer scars, which bring them closer to burn scars, may explain why they are particularly affected by carcinomatous degeneration. One could also mention the chronicity of the wound in this infection, or wonder if the mycobacteria itself could play a role in carcinogenesis. This observation is, in our opinion, an alarm signal. We must fear an outbreak of cases in the years to come. To this end, preventive measures should already be taken by sensitizing the patients for an early consultation before any modification of their scars. After recovery, Buruli ulcer seems to present a risk of long-term evolution to a cancer. The scars of this condition, which could be considered precancerous lesions.

## Introduction

Buruli ulcer is infectious necrotizing panniculitis due to *Mycobacterium ulcerans* [1]. Currently, the endemic continues to grow and his effect is increasing dramatically, especially in West African countries such as Côte d'Ivoire. Buruli ulcer is distinguished by its chronic evolution, characterized by extensive skin rashes complicated by dystrophic, fibrous and retractile scars [2-4]. On these scars can occur in the long term a formidable complication, squamous cell carcinoma. In Abidjan, the first case was observed in 2010 [5], then eight cases were observed in 2015 [1]. In Bouake, a city in the center of Ivory Coast, we describe the first case of squamous cell carcinoma occurring on cicatrization of Buruli ulcer in a 32-year-old patient with no comorbidities.

## Patient and observation

**Figure 1:**
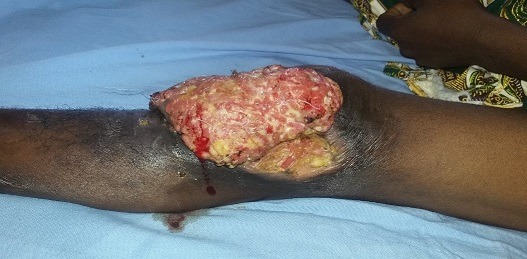
Burgeoning ulcer tumor

**Figure 2:**
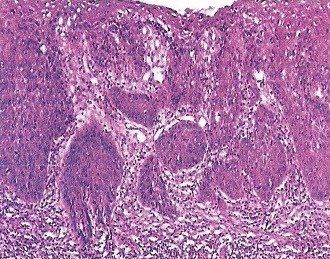
Histological appearance of squamous cell carcinoma

A 32-year-old HIV-negative patient consults the department for an ulcer-budding swelling in her left knee that has been evolving for 2 months. The antecedents are marked by a bifocal Buruli ulcer (knee and hand) cured for about 10 years leaving room for a retractile and deforming limb scar. The examination revealed a large swelling of about ten centimeters in diameter, ulcero-budding with a cauliflower aspect bleeding easily on contact with the sore edges, painful, sitting on the outer anterolateral aspect of the knee left. The peri-lesional skin was normal in appearance (Figure 1). Inguinal lymphadenopathy was noticeable, moving about two centimeters in diameter. Biology has found hypochromic normocytic anemia. Histology has revealed a proliferation of atypical squamous cells (large hyperchromatic nuclei, numerous mitoses) in invasive lobules, associated with disorders of keratinization. Finally the tumor stroma is inflammatory (Figure 2). X-ray of the knee showed bone lysis. The diagnosis of squamous cell carcinoma being retained without metastasis, resection of the tumor with scarring after one month without chemotherapy. There was no recurrence after six months of decline.

## Discussion

Buruli ulcer is hyper-endemic in West and Central Africa: 16,517 cases were recorded from 2006 to 2015 and each year, about 500 new cases are listed in Côte d'Ivoire which remains a very active focus [4]. The epidemiology of the infection responsible for the scars [6, 7] explains the young age of our patient and the localization of the carcinoma on the limb. No comorbidity, including HIV infection, which is a factor favoring the development of malignant tumors in sub-Saharan Africa, has been noted in our observation. The healing of the lesions occurs after several months of evolution. In our report, our patient had retractile and fibrous scars after healing. The occurrence of cancers on Buruli ulcer scars is not known. Isolated cases of squamous cell carcinoma have already been described [8, 9].

The first Ivorian observation was reported in 2010 [5]. Since then, eight cases have been recruited by the Abidjan center, which suggests a higher figure nationally since the center of Abidjan does not have a monopoly on the management of ulcer disease Buruli. While exposure to the sun is the main risk factor for skin squamous cell carcinoma in light-skinned individuals, non-sun factors are thought to be involved in people with pigmented skin. These are mainly chronic leg ulcers (neglected posttraumatic or infectious), HIV infection, discoid lupus and various chronic scars [10, 11]. The carcinomatous degeneration of scars, especially the scars of old burns, is constantly reported. There is a lack of epidemiological studies on this topic in the countries of northern Africa and sub-Saharan Africa where sunshine is high, medical resources are limited, and the risk of repeated ulcerations of scars becomes higher. The etiology of cancers occurring on scars is not fully understood, although current assumptions include proliferation due to chronic inflammation and tissue irritation. Also, permanent tissue exposure to toxins and co-carcinogenic factors after injury, as well as poor vascularization of scar tissue, weaken local immune defenses [12, 13]. The characteristics of Buruli ulcer scars, which bring them closer to scars of burns, may explain why they are particularly the cause of carcinomatous degeneration. One could also mention the chronicity of the wound in this infection, or wonder if the mycobacteria itself could play a role in carcinogenesis.

This observation is, in our opinion, an alarm signal. Given the number of people affected by this disease in their childhood or adolescence in Côte d'Ivoire and more generally in sub-Saharan Africa, there is a fear of an outbreak of cases in the years to come when these adolescents have reached adulthood. To this end, preventive measures should now be taken in the countries concerned: instituting systematic surveillance of patients “cured” of Buruli ulcer in order to detect the first signs of carcinomatous degeneration and to sensitize patients for early consultation before any modification of their scars. Indeed, earlier management (at a stage without bone involvement or metastasis) of these cases could have improved their prognosis.

## Conclusion

After healing, Buruli ulcer seems to present a risk of long-term progression to cancer. This risk needs to be better assessed but it is already important to monitor the scars of this condition, which could be considered precancerous lesions.

## Competing interests

The authors declare no competing interests.
